# Invasive lobular breast carcinoma with diffuse gastrointestinal metastases: a visual diagnostic pitfall

**DOI:** 10.1093/oncolo/oyag107

**Published:** 2026-03-24

**Authors:** Jerry H Rose, Shaya Noorian, Nima Sharifai, Eric M Goldberg

**Affiliations:** Department of Medicine, Division of Gastroenterology and Hepatology, University of Maryland School of Medicine, Baltimore, MD 21201, United States; Department of Medicine, Division of Gastroenterology and Hepatology, University of Maryland School of Medicine, Baltimore, MD 21201, United States; Department of Pathology, University of Maryland School of Medicine, Baltimore, MD, United States; Department of Medicine, Division of Gastroenterology and Hepatology, University of Maryland School of Medicine, Baltimore, MD 21201, United States

**Keywords:** invasive lobular carcinoma, breast cancer metastases, secondary gastrointestinal malignancy, endoscopy

## Abstract

Invasive lobular carcinoma (ILC) of the breast demonstrates a distinct infiltrative growth pattern and a predilection for metastasis to the gastrointestinal tract, often without forming discrete masses. We report on a 64-year-old woman with a history of estrogen receptor–positive, human epidermal growth factor receptor 2-negative ILC who presented with progressive nausea, vomiting, and diarrhea. Recent surveillance imaging had shown no evidence of metastatic disease. Computed tomography revealed nonspecific findings despite extensive mucosal involvement that was later identified on endoscopy. Upper endoscopy and colonoscopy demonstrated diffuse nodular and congested mucosa throughout the duodenum and colon without focal lesions. Histologic evaluation revealed poorly cohesive tumor cells infiltrating the lamina propria, and immunohistochemistry was positive for cytokeratin 7, GATA3, and estrogen receptor, confirming metastatic breast carcinoma. Diffuse gastrointestinal involvement by ILC is uncommon and may mimic inflammatory, infectious, ischemic, or medication-related conditions, contributing to delayed diagnosis. This case highlights an important diagnostic pitfall and underscores the need to consider metastatic disease in patients with a history of lobular breast carcinoma who present with unexplained gastrointestinal symptoms, even in the absence of radiographically apparent disease.

A 64-year-old woman with a history of estrogen receptor–positive, progesterone receptor–negative, and human epidermal growth factor receptor 2 (HER2)-negative invasive lobular carcinoma of the left breast presented with several months of intractable nausea, vomiting, and diarrhea. She had previously received neoadjuvant dose-dense doxorubicin and cyclophosphamide followed by paclitaxel, left partial mastectomy with axillary lymph node dissection, and adjuvant whole-breast and nodal radiation therapy, and was maintained on endocrine therapy with anastrozole and the cyclin-dependent kinase 4/6 inhibitor abemaciclib, which was discontinued after 1 year because of diarrhea. Surgical pathology demonstrated residual invasive lobular carcinoma (ILC) with 8 of 16 positive lymph nodes. At a routine oncology follow-up 1 month prior to presentation, physical examination and surveillance positron emission tomography/computed tomography (PET/CT) imaging showed no evidence of recurrent or metastatic disease. She subsequently developed progressive gastrointestinal symptoms, prompting further evaluation. Laboratory studies demonstrated anemia (hemoglobin 8.7 g dL^−1^; reference range 12-15.7 g dL^−1^), elevated transaminases (aspartate aminotransferase 130 U L^−1^ and alanine aminotransferase 55; reference 0-36 U L^−1^), and hyperbilirubinemia (total bilirubin 1.7 mg dL^−1^; reference 0.3-1.2 mg dL^−1^). Computed tomography of the abdomen and pelvis revealed gallbladder distention with common bile duct dilation and slight terminal ileum thickening, findings that were otherwise nonspecific and did not reflect the extent of mucosal disease later identified endoscopically.

Upper endoscopy and colonoscopy demonstrated a diffusely nodular, congested mucosa involving the duodenum and extending throughout the colon (Figure 1). No discrete mass lesions were identified. Biopsies obtained from multiple sites revealed carcinoma infiltrating the lamina propria. Histologic evaluation demonstrated poorly cohesive tumor cells infiltrating the lamina propria between intestinal crypts (Figure 2A–C). Immunohistochemical staining showed tumor cell positivity for cytokeratin 7, GATA-binding protein 3, and estrogen receptor, with negative staining for cytokeratin 20, supporting metastatic breast carcinoma (Figure 2D–G). Repeat breast biomarker analysis demonstrated estrogen receptor positivity with progesterone receptor and HER2-negativity, consistent with the patient’s known ILC.

**Figure 1.**
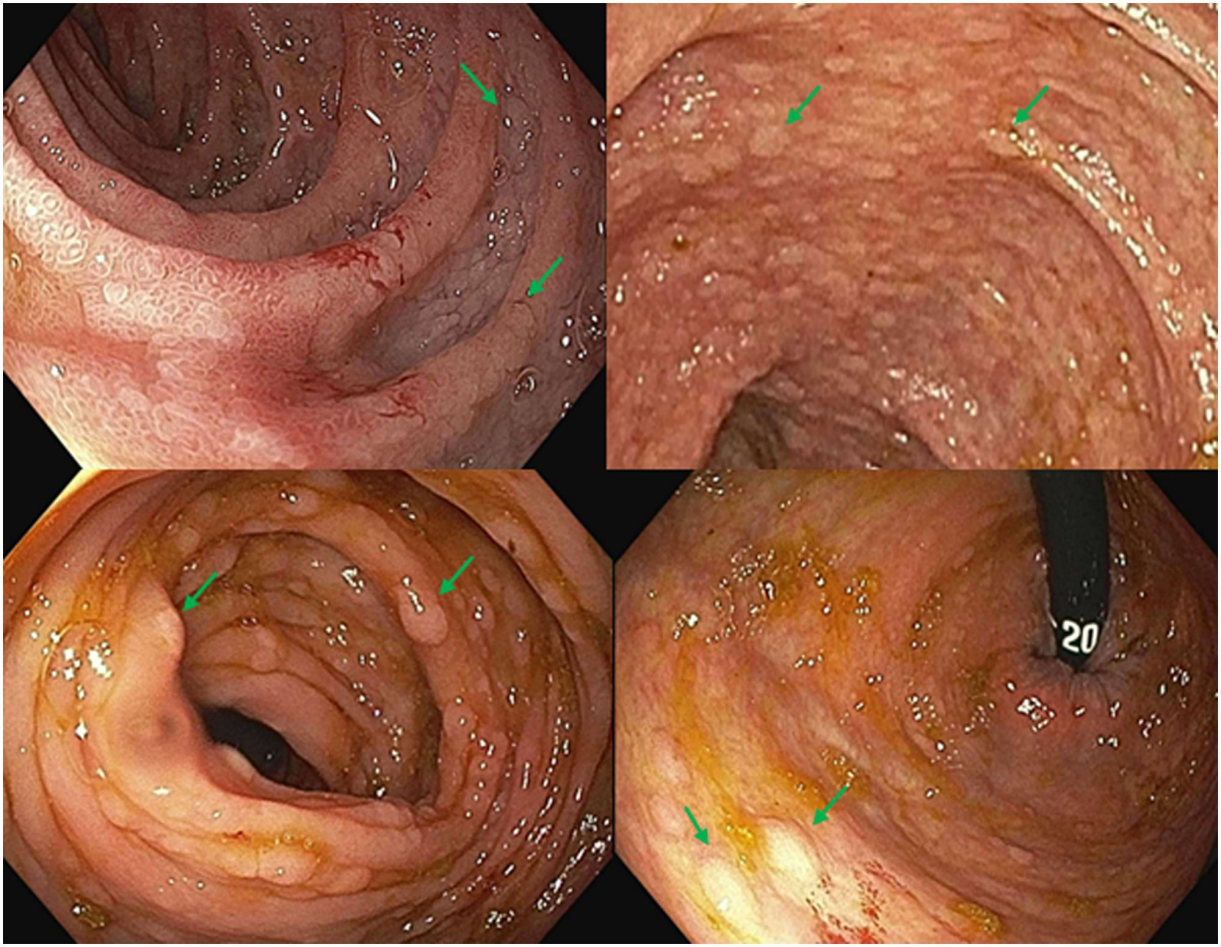
Colonoscopy reveals diffusely nodular and congested colonic mucosa.

**Figure 2.**
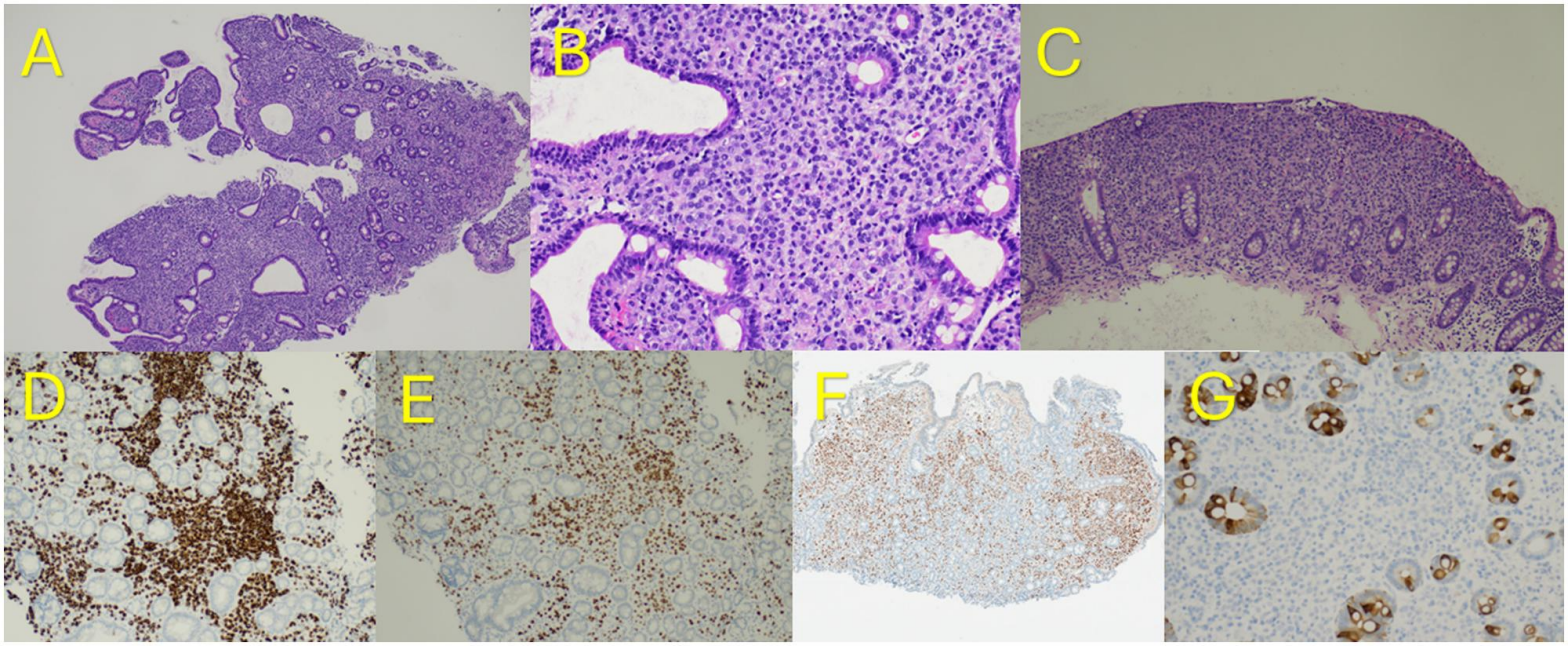
Hematoxylin and eosin sections of duodenal mucosa (A, 40× original magnification; B, 200×) and colonic mucosa (C, 40×) show an infiltrative cellular population expanding the lamina propria between intestinal crypts. The neoplastic cells are poorly cohesive, with round nuclei and modest amounts of cytoplasm. Tumor cells show immunoreactivity for CK7 (D), GATA3 (E), and ER (F), and are negative for CK20 (G), supporting a metastatic breast origin.

ILC is characterized by a unique infiltrative growth pattern and has a recognized propensity for metastasis to the gastrointestinal tract, peritoneum, and retroperitoneum.^1^^,^^2^ Unlike ductal carcinoma, lobular carcinoma frequently spreads in a diffuse, non–mass-forming manner, which may result in subtle endoscopic and radiographic findings. Diffuse luminal involvement of both the small intestine and colon remains uncommon (4.5% of ILC cases) and may closely mimic inflammatory, infectious, ischemic, or medication-related gastrointestinal conditions.^3–5^ Infectious etiologies such as cytomegalovirus colitis, *Mycobacterium avium* complex infection, and intestinal tuberculosis, as well as medication-induced colitis resulting from nonsteroidal anti-inflammatory drugs, antibiotics, or immunotherapies, may produce similar nonspecific mucosal abnormalities. Endoscopically, this pattern may also resemble inflammatory bowel disease, lymphoproliferative disorders, or diffuse infiltrative malignancies. The absence of discrete mass lesions and the presence of subtle mucosal nodularity highlight the importance of maintaining suspicion for metastatic disease and obtaining biopsies even when findings appear nonspecific. As a result, diagnosis is often delayed unless random or targeted biopsies are pursued.

This case highlights an important diagnostic pitfall for oncologists and gastroenterologists alike. In patients with a history of invasive lobular breast carcinoma who present with unexplained gastrointestinal symptoms, diffuse metastatic involvement of the gastrointestinal tract should be considered even in the absence of focal lesions. Recognition of this metastatic pattern is critical, as early histologic confirmation can prevent misdiagnosis and guide appropriate oncologic decision-making. Despite supportive care and antiemetics, the patient’s symptoms progressed, and she was referred to palliative oncology for management of advanced metastatic disease.

## Conflicts of interest 

None declared.

## Previous presentation

This article was not presented at a professional meeting.

## Statement on informed consent

Written informed consent for publication of clinical details and images was obtained from the patient.

## Data Availability

The data supporting the findings of this study are available within the manuscript. No additional data are available.
